# Differential impacts of cardiac and abdominal ectopic fat deposits on cardiometabolic risk stratification

**DOI:** 10.1186/s12872-016-0195-5

**Published:** 2016-01-22

**Authors:** Fu-Zong Wu, Carol C. Wu, Pei-Lun Kuo, Ming-Ting Wu

**Affiliations:** Department of Radiology, Kaohsiung Veterans General Hospital, No. 386, Ta-Chung 1st Road, Kaohsiung, 81362 Taiwan; Institute of Clinical Medicine, National Yang Ming University, Taipei, Taiwan; Department of Radiology, University of Texas MD Anderson Cancer Center, Houston, TX USA

**Keywords:** Ectopic fat deposits, Cardiometabolic risks

## Abstract

**Background:**

Previous studies have shown that excessive abdominal visceral adipose tissue (AVAT) and epicardial adipose tissue (EAT) are risk factors of cardiometabolic disease; we hypothesized there is differential contribution of abdominal and cardiac fat deposits to the cardiometabolic profiles.

**Methods:**

Two hundred eight consecutive subjects with clinical suspicion of coronary artery disease (CAD) who underwent cardiac and abdominal CT for Agatston score and abdominal visceral fat measurement were retrospectively analyzed. Regional thickness of EAT (EATth), total volume of EAT, total volume of paracardial adipose tissue (PAT) and total volume of AVAT from L2 to L5 level were measured. The relationships between abdominal and cardiac adipose tissue measurements, the number of components of metabolic syndrome, and the severity of Agatston score on a four ranking scale (0, 1–10,11–100, 101–400, >400) were investigated.

**Results:**

The amounts of AVAT, EAT, PAT and EATth-LAVG showed a significant linear trend with increasing number (0–5) of components in metabolic syndrome (AVAT, EAT and PAT P for trend <0.0001; EATth-LVAG P for trend <0.001). EATth at left atrioventricular groove (EATth-LAVG) showed significant linear trend with the severity of Agatston score on a four ranking scale (P for trend <0.0001). In multivariate binary regression analysis, total volume of AVAT was the sole adiposity predictor for metabolic syndrome independent to age, gender, and waist circumference (odds ratio of 1.20, 95 % CI 1.08–1.32, *p* < 0.001) while total volume of EAT, PAT, and EATth-LAVG were not. In contrary, EATth-LAVG was the sole adiposity predictor for Agatston score >400 (odds ratio of 1.11, 95 % CI 1.034–1.184, *p* = 0.004).

**Conclusions:**

Excessive total volume of AVAT appears to be preferentially associated with metabolic syndrome; while EAT, esp. EATth-LAVG is preferentially associated with coronary artery disease. This differential effect of the two adiposities deserves a large-scale cohort study for further investigation.

## Background

Visceral adiposity has been increasingly recognized as a well-established risk marker of cardiometabolic disease [1–4]. Visceral adiposity, as an endocrine organ, secretes different adipokines, including cytokines and chemokines, which can have systemic effects [5–7]. Distribution of ectopic fat is considered as an important predictor of cardiometabolic risks. Different distributions of excessive ectopic fat deposits in various locations including the liver, muscle, kidneys, heart, subcutaneous region and abdomen may be associated with differential impacts on cardiometabolic risks [5, 8]. These site-specific differences of ectopic fat depots are therefore important in understanding the role of visceral adiposity as a causal factor in cardiometabolic risks. Previous studies have highlighted the association between abdominal visceral fat and cardiometabolic risks [9–11], abdominal subcutaneous fat and metabolic syndromes [10, 12, 13], epicardial adipose tissue (EAT) adiposity and CAD risks [14–16], and regional-specific EAT adiposity with cardiometabolic risks [17, 18]. Abdominal visceral fat is believed to be intrinsically different from cardiac visceral fat. Both excessive abdominal adiposity and cardiac adiposity are essential contributors to the development of the cardiometabolic risks. Although they tend to occur together, their quantitative relationship and their respective roles in various cardiometabolic components remain unclear. To the best of our knowledge, none of these studies provided comprehensive quantitative estimates of the relative contribution to the cardiometabolic risks of abdominal fat (including abdominal visceral fat and abdominal subcutaneous fat) and cardiac fat (including epicardial, pericardial and regional-specific cardiac fat). There is thus a lack of studies specifically aiming at systematic investigation, using precise measurement techniques, of the relative importance of excessive abdominal and cardiac fat accumulation in explaining the variation of the components of the cardiometabolic risks. Our work is the first to systematically investigate the impacts of the differential ectopic fat deposits on cardiometabolic risks. In addition, different types of regional-specific or total volume of EAT depot may play differential roles in the progression of coronary artery atherosclerosis; however, this issue has not been well addressed [16, 18–20].

## Methods

### Study populations

A total of 208 consecutive subjects with clinical suspicion of CAD underwent EKG-gated coronary artery calcification (CAC) scanning and non-contrast enhanced abdominal CT from January 2007 to June 2010 at our institution were retrospectively studied. Complete medical histories including body mass index (BMI), presence of hypertension and/or diabetes mellitus, smoking history and waist circumstance were recorded for all participants. Blood levels of high-density lipoprotein-cholesterol (HDL-C) and serum triglyceride (TG) concentration were also recorded. The study protocol was approved by the Institutional Review Board Committee of Kaohsiung Veterans General Hospital, Kaohsiung, Taiwan. The requirement for informed consent was waived.

### CAC-scoring CT and assessment of Agatston score

CT scan was performed on a 64-detector raw CT scanner (Aquilion 64, Toshiba Medical Systems, Tokyo, Japan) with the following parameters: 64 × 0.5-mm collimation, rotation time 350 ms, pitch factor 0.2–0.3, tube voltage 120 kV, tube current 400 mA. Beta-adrenergic antagonist (Metoprolol 50–100 mg, AstraZeneca, Hertfordshire, England) was orally administered 60 min before a scan if the patient’s heart rate ≥70 beats/min. The Agatston scores were calculated using standard quantification algorithms with semiautomatic software (Vitrea 2; Vital Images, Minnetonka, MI, USA). Agatston scores were further categorized into four ranks (0, 1–100, 101–400, and >400) for CAD risk stratification.

### Measurement of abdominal and cardiac adipose tissue by CT

**Fig. 1:**
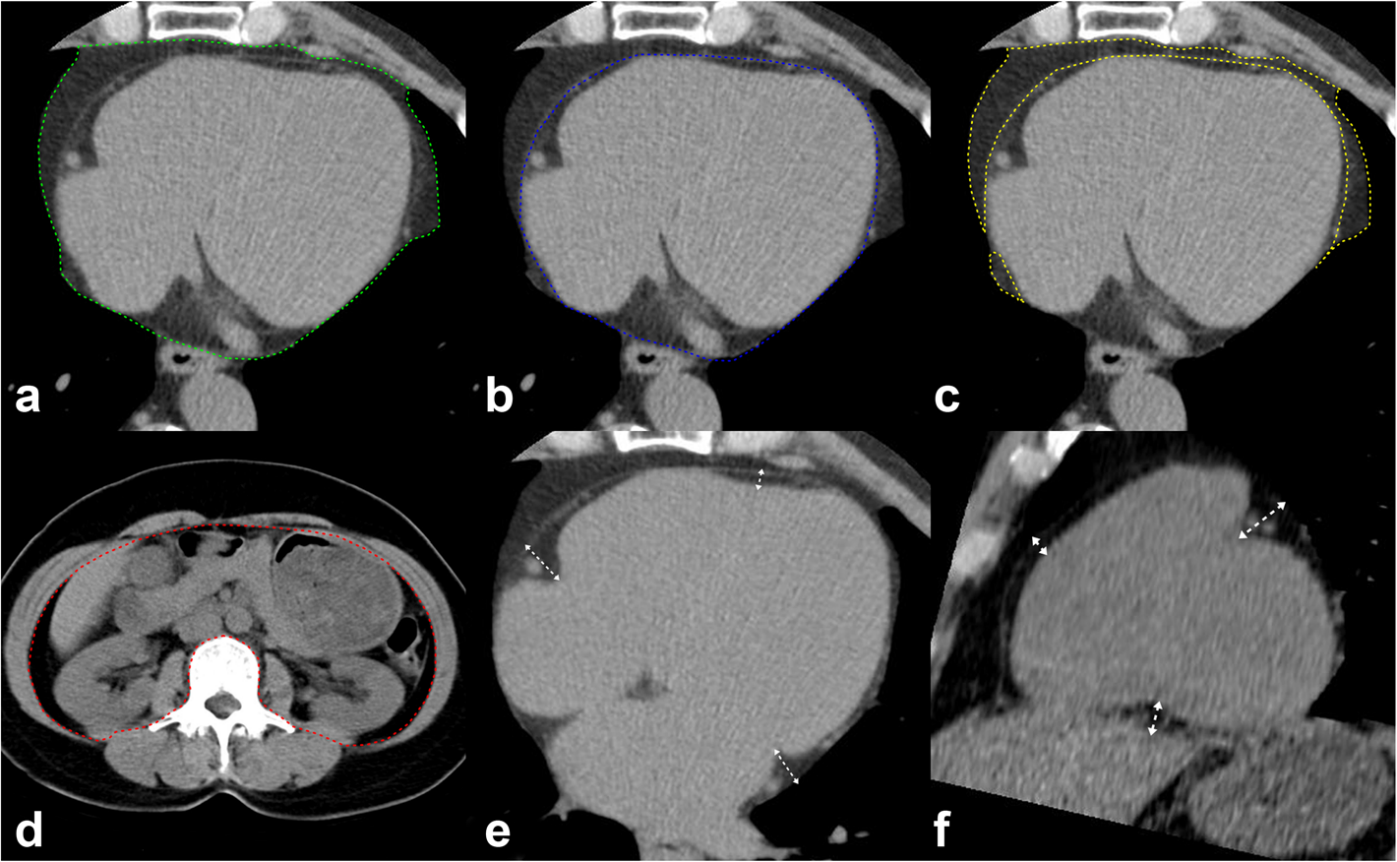
Quantification of different abdominal and cardiac fat deposits by CT with a threshold of −200 to −50 HU on a 3-dimensional workstation. **a** Measurement of total pericardial fat volume by manual tracing (*green boundary*) of the area of the pericardial fat from the left main pulmonary artery level to the left ventricular apex. **b** Measurement of total volume of EAT was performed on axial images by manual tracing (*blue boundary*) of the parietal pericardium from the left main pulmonary artery level to the left ventricular apex. **c** Total volume of PAT (*yellow boundary*) = total pericardial fat volume (PAT + EAT) – total EAT volume. **d** Measurement of total volume of AVAT was performed on axial images by manual tracing (*red boundary*) of the parietal peritoneum from L2 to L5 levels. **e** Regional EAT thickness was assessed as the distance from myocardium in the orthogonal direction to visceral epicardium on the horizontal long-axis plane at the left AVG, right AVG, and anterior IVG. **f** On the parasternal short-axis plane, regional EAT thickness was measured at superior IVG, inferior IVG, and right ventricular (RV) free wall. Double-headed arrows indicate the measurements of the EAT thickness. HU = Hounsfield units; EAT = epicardial adipose tissue; PAT = paracardial adipose tissue; AVAT = abdominal visceral adipose tissue; AVG = atrioventricular groove; IVG = interventricular groove; RV = right ventricle

Abdominal (including abdominal subcutaneous fat and abdominal visceral fat) and cardiac (including epicardial fat volume, pericardial fat and regional epicardial fat thickness) fat measurements were all performed on a workstation (Advantage Workstation 4.3, GE Healthcare) as described in our previous studies shown in Fig. 1(a-f) [21]. Adipose tissue was defined as Hounsfield units between −50 and −200, as previously reported [22]. Total volume of abdominal visceral adipose tissue (AVAT) was measured on axial images with a 0.5 mm slice thickness at 8 mm intervals starting from L2 to L5 levels in each subject. EAT was defined as adipose tissue located within the pericardial sac. Paracardial adipose tissue (PAT) was defined as adipose tissue located outside the pericardial sac. Total volume of EAT and PAT were measured on axial images starting from the level of left main pulmonary artery to the left ventricular apex with about 35 cross-section slices. EAT thickness was measured at the left atrioventricular (AV) groove, right AV groove and anterior AV groove, and abbreviated as EATth-LAVG, EATth-RAVG and EATth-AIVG respectively. Maximal fat thickness assessed as the distance from myocardium to visceral epicardium in the orthogonal direction was determined [23]. Details regarding measurements of EAT thickness and reproducibility for all EAT measurements were described previously [21].

### Characteristics of metabolic syndrome component*s*

The metabolic syndrome components were defined according to the criteria described by the National Cholesterol Education Program Adult Treatment Panel III [24], including (1) Presence of the abdominal obesity (waist circumference ≥102 cm and ≥88 cm in men and women, respectively); (2) elevated blood pressure (SBP ≥130 mmHg and/or DBP ≥85 mmHg); (3) low HDL-C (<40 mg/dl and <50 mg/dl in men and women, respectively); (4) TG ≥150 mg/dl, and (5) fasting plasma glucose ≥100 mg/dl (or current diabetic medication). All five components of the metabolic syndrome were assessed, and metabolic syndrome is diagnosed when a patient has at least three of the five criteria.

### Statistical analysis

The data for the continuous variables with a normal distribution are expressed as means ± SDs and the data for the continuous variables without a normal distribution are expressed as medians with interquartile ranges.

To assess linear trends in various abdominal and cardiac adipose tissue measurements for increasing numbers of metabolic syndrome components and the severity of Agatston score, the general linear model controlling for age and gender was used to test the linear trend of measurements of abdominal and cardiac fat according to the number of metabolic syndrome components and the severity of Agatston score.

To determine different impacts of various abdominal or cardiac adipose tissue measurements on cardiometabolic risks, principal component analysis was used.

The resulting factor pattern was interpreted by using factor loadings, and the most powerful factors (eigen values >1.0) were retained for further analysis. To interpret the results from factor analyses, the pattern of factor loadings was examined to determine which original variables represent primary constituents of each factor. An absolute loading value ≥0.40 was used to interpret the resulting factor pattern.

Multivariate logistic regression analysis was used to determine the relationships between the metabolic syndrome with abdominal and cardiac adiposity measurements, including the total volume of EAT, PAT, AVAT and EATth-LAVG after adjustment for age, gender and waist circumference. Multivariate logistic regression analysis was used to determine the relationships between the severity of Agatston score with abdominal and cardiac adiposity measurements, including the total volume of EAT, PAT, AVAT and EATth-LAVG after adjustment for age, gender, waist circumference, BMI, hypertension, diabetes mellitus, HDL-C, triglycerides and current smoker.

## Results

### Clinical characteristics of the study participants

We included 208 subjects (mean age 54.94 years,79 % male) with detailed baseline characteristics and various adipose tissue measurements demonstrated in Table 1. The BMI and waist circumference were 24.90 ± 3.36 kg/m2 and 89.17 ± 8.90 cm respectively. 45 % were current smokers; 40 % had hypertension and 23 % had diabetes mellitus.

**Table 1:** Clinical characteristics and measurements of various adipose tissues in study participants (*n* = 208)

Age (years)	54.94 ± 9.05
Men (male)	79 %
Waist circumference (cm)	89.17 ± 8.90
BMI (kg/m2)	24.90 ± 3.36
HDL-C (mg/dL)	41 (18, 201)
Triglycerides (mg/dL)	130 (44, 2182)
Diabetes mellitus (%)	23 %
Current smokers (%)	45 %
Hypertension (%)	40 %
Number of metabolic components	2.35 ± 1.32
Agatston score	8.50 (44,2182)
Total volume of AVAT (mm^3^)	1129.09 ± 530.62
Total volume of PAT (mm^3^)	219.29 ± 90.02
Total volume of EAT (mm^3^)	121.33 ± 45.65
EATth-LAVG (mm)	15.92 ± 4.47
EATth-RAVG (mm)	17.08 ± 3.74
EATth-AIVG (mm)	8.36 ± 10.96

Data are presented as mean ± SD or median (min; max), depending on distribution

*BMI* body mass index, *HDL* high density lipoprotein, *AVAT* abdominal visceral adipose tissue, *SAT* abdominal subcutaneous adipose tissue, *EAT* epicardial adipose tissue, *PAT* paracardial adipose tissue, *EATth-LAVG* EAT thickness at left AV(atrioventricular) groove, *EATth-RAVG* EAT thickness at right AV(atrioventricular) groove, *EATth-AIVG* EAT thickness at anterior IV (interventricular) groove

### Correlations between cardiometabolic risk and adipose tissue measurements

**Fig. 2:**
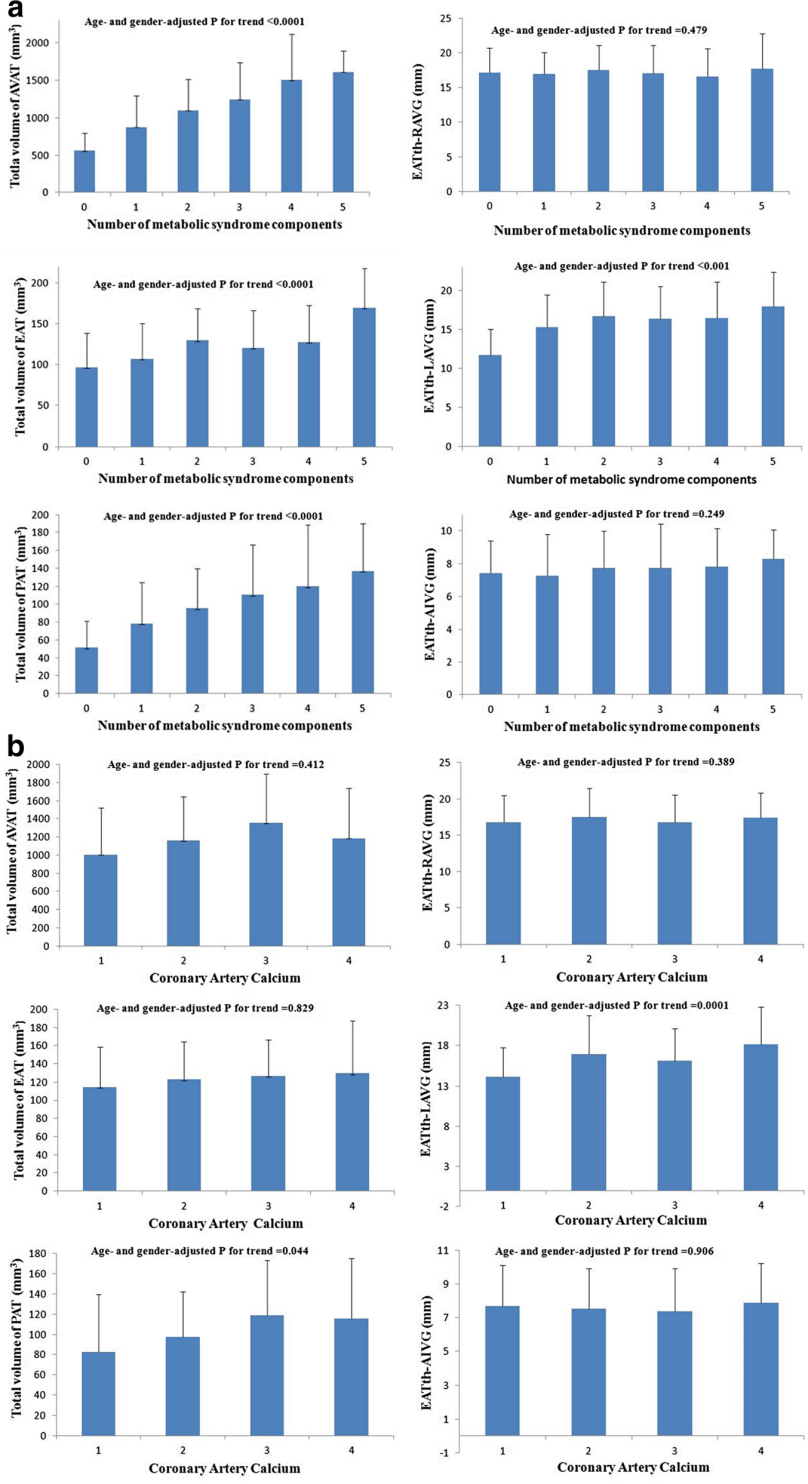
**a** The general linear model was used to test the linear trend of measurements of abdominal and cardiac fat according to the number of metabolic syndrome components. The volumetric and thickness measurement of abdominal and cardiac fat in relation to the number of metabolic syndrome components (P for trend <0.0001 in the AVAT, PAT, and EAT group; P for trend <0.001 in the EATth-LAVG group). **b** The general linear model was used to test the linear trend of measurements of abdominal and cardiac fat according to the Agatston score on a four ranking scale. The volumetric and thickness measurement of abdominal and cardiac fat in relation to coronary artery calcium (Agatston score on a four ranking scale, P for trend <0.0001 in the AVAT, PAT, and EAT group; P for trend <0.0001 in the EATth-LAVG group)

As shown in Fig. 2a, total volume of AVAT, EAT, PAT and EATth-LAVG significantly increased as the number of the metabolic syndrome components increased (p for trend <0.001), whereas all other CT measurements of epicardial thickeness including EATth-RAVG and EATth-AIVG did not. On the other hand, the only EATth-LAVG was significantly increased with the severity of Agatston score on a four ranking scale (*p* for trend <0.001), whereas all other CT measurements including total volume of AVAT, EAT, and PAT, EATth-RAVG and EATth-AIVG did not demonstrate significant correlation (Fig. 2b).

### Pearson correlation coefficients between cardiometabolic risk and adipose Tissue measurements

As shown in Table 2, total volume of AVAT, EAT, PAT and EATth-LAVG were more positively and significantly related to BMI, waist circumference and metabolic syndrome components, whereas the EATth- RAVG and EATth- AIVG were not. Among the correlations between metabolic syndrome components and various measurements of abdominal and cardiac adipose tissue, we found the total volume of AVAT had the best correlation (*r* = 0.519), followed by the total volume of EAT (*r* = 0.382), the total volume of PAT (*r* = 0.264), and EATth-LAVG (*r* = 0.250) (all *p* < 0.001).

**Table 2:** Correlation between various cardiac and abdominal adipose tissue measurements and cardiomeatbolic profiles

	BMI	Waist circumference	Metabolic syndrome components	Agatston score on a four ranking scale	Agatston score
Total volume of AVAT	0.706^**^	0.775^**^	0.519^**^	0.169^*^	0.064
Total volume of PAT	0.529^**^	0.629^**^	0.382^**^	0.250^**^	0.109
Total volume of EAT	0.487^**^	0.526^**^	0.264^**^	0.127	0.083
EATth-LAVG	0.275^**^	0.239^**^	0.250^**^	0.307^**^	0.287^**^
EATth-RAVG	0.059^**^	0.116	−0.014	0.046	0.100
EATth-AIVG	0.052^**^	0.074	0.052	−0.060	−0.019

*Abbreviation*: as Table 1

^*^ *p* <0.01

^**^ *p* < 0.001

Among all the EAT measurements, the EATth-LAVG had the most significant correlation with BMI (*r* = 0.487, *P* < 0.001) and metabolic syndrome components. The total volume of AVAT and PAT had only weak correlation with Agatston score ranking.

Among the correlations between Agatston socre and various measurements of abdominal and cardiac adipose tissue, we found the EATth-LAVG had the best correlation (*r* = 287, *P* < 0.001), whereas the other CT measurements including EATth-RAVG and EATth-AIVG had no significant correlation.

#### Clustering of cardiometabolic risk factors: factor analysis

Table 3 displays the results of factor analysis of core cardiometabolic variables among 208 subjects. The factor-loading pattern of the two factors (components) identified in the study is presented in Table 3. More importantly, the factor 1 and factor 2 cumulatively explained 65.01 % of the total variation of cardiometabolic risk in the study.

**Table 3:** Factor analysis of different ectopic visceral adiposity and cardiometabolic risks

Characteristic	Factor 1	Factor 2
Waist circumference	0.881	−0.156
BMI	0.841	−0.228
Metabolic components	0.570	0.188
Agatston score	0.157	0.823
Total volume of AVAT	0.890	−0.136
Total volume of PAT	0.809	0.059
Total volume of EAT	0.702	−0.046
EATth-LAVG	0.467	0.629
Percentage of total variance explained	49.91	15.10
Cumulative %	49.91	65.01

Factor loading above 0.5 (positive or negative) are considered high

*Abbreviation*: as Table 1

Factor 1 had strong contributions from BMI, waist circumference, metabolic syndrome components, total volume of AVAT, EAT and PAT. This factor was interpreted as a “volumetric abdominal or cardiac adiposity-metabolic factor” and explained 49.91 % of the total variance. Factor 2 had strong contributions from only two components including Agatston score and EATth-LAVG. This factor was interpreted as a “regional-specific cardiac adiposity-CAD factor” and explained 15.10 % of the total variance.

#### Abdominal or cardiac adiposity in cardiometabolic risk factors

Table 4 shows the multivariate logistic regression analysis to determine the predictors of metabolic syndrome. Total volume of AVAT is the most important sole independent predictor of metabolic syndrome after adjusting for age, gender and waist circumference (odds ratio of 1.20, 95 % CI 1.08–1.32, *p* < 0.001). In multivariate ordinal logistic regression analysis, EATth-LAVG is an independent predictor (odds ratio of 1.11, 95 % CI 1.034–1.184, *p* = 0.004) for Agatston score >400 after adjusting for age, gender, waist circumference, BMI, hypertension, diabetes mellitus, HDL-C, triglycerides and current cigarette use while other measurements of abdominal or cardiac adipose tissue are not (summarized in Table 5).

**Table 4:** Multivariate binary logistic regression analysis for predictors of presence of metabolic syndrome

Predictor^a^	OR	95 % CI	*P* value
Age (years)	1.039	0.996–1.084	.074
Gender (male)	0.196	0.063–0.614	.154
Waist circumference (cm)	1.010	0.963–1.067	.051
Total volume of AVAT (mm^3^)	1.200	1.080–1.320	<0.001
Total volume of PAT (mm^3^)	1.002	0.991–1.013	.747
Total volume of EAT (mm^3^)	0.995	0.984–1.006	.378
EATth-LAVG (mm)	0.954	0.871–1.046	.316

**Table 5:** Multivariate binary logistic regression analysis for predictors of Agatston score > 400 or not

Predictor^a^	OR	95 % CI	*P* value
Age (years)	1.10	1.065–1.143	<0.001
Gender (male)	0.18	0.070–0.496	.001
Waist circumference (cm)	1.02	0.966–1.070	.528
Hypertension	0.56	0.314–0.979	.059
Diabetes mellitus	0.46	0.237–0.912	.026
HDL-C (mg/dL)	0.99	0.979–1.018	.858
Triglycerides (mg/dL)	1.00	0.998–1.002	.863
Current smokers	0.93	0.508–1.707	.819
Total volume of AVAT (mm^3^)	0.99	0.998–1.000	.303
Total volume of PAT (mm^3^)	0.99	0.999–1.015	.088
Total volume of EAT (mm^3^)	0.99	0.986–1.002	.142
EATth-LAVG (mm)	1.11	1.034–1.184	.004

Dependent variable: ^a^ Adjusted for conventional CAD risk factors (age, gender, diabetes mellitus, hypertension, smoking habit, waist circumstance, HDL-C and serum triglyceride) and Agatston score. CAD: coronary artery disease; others, see Table 1

## Discussion

### Main findings of the study

Many previous studies have investigated the relationships between abdominal fat (visceral or subcutaneous) and cardiometabolic risk factors [9, 10, 12, 13], epicardial fat and cardiometabolic risk factors [14, 16, 17], epicardial fat and subclinical atherosclerosis [25], intra-hepatic fat and cardiometabolic risk factors and epicardial fat with CAC score [9, 16, 26–28]. However, none of these studies comprehensively examined the relative contributions of abdominal fat (including visceral subcutaneous fat) and cardiac fat (including epicardial, pericardial and regional-specific cardiac fat) on the cardiometabolic risks. This is the first study to investigate different distribution of abdominal and cardiac fat and to correlate the amount fat deposits with variously cardiometabolic risks simultaneously. Our study demonstrated that the total volume of AVAT most strongly correlates with the number of metabolic syndrome components, BMI and waist circumference, whereas cardiac fat, especially EATth-LAVG, is strongly associated with the severity of Agatston score. These findings imply that AVAT may contribute to metabolic syndrome, while regional-specific cardiac adipose tissue (EATth-LAVG) may contribute to coronary atherosclerosis, measured by the severity of Agatston score. Furthermore, the present findings also confirm previous evidences that ectopic fat depots including VAT, intrahepatic fat, and intramuscular fat with predominantly potential systemic effects and associated with increased metabolic risk. In contrast, ectopic fat depots including pericardial and perivascular fat are postulated to have a predominantly potential local toxic effect with increased cardiovascular risks [5, 8]. In addition, our study further demonstrated that regional-specific cardiac adipose tissue may predisposes to atherosclerosis measured by Agatston score on a four ranking scale. This finding is also supported by our previous study and other studies [18, 21, 29].

### Global or regional adiposity in cardiometabolic risk factors

Recent studies have shown independent effects of global visceral adipose tissue on cardiometabolic risks [9, 10, 30], and other authors have shown that increased regional or peri-coronary EAT predicts both cardiovascular disease and metabolic syndrome [16–18]. In our study, clusters of cardiometabolic risk factors and various measurements of adipose tissue were identified by factor analysis. We defined the term The factor 1(defined as volumetric abdominal or cardiac adiposity-metabolic factor, and be more representative of global fat-metabolic factor) explained the maximum variance (49.91 %) and is in concordance with the fact that global adipose tissues including total volume of AVAT, EAT and PAT are more associated metabolic syndrome components, BMI and WC. Among these parameters, total volume of AVAT showed the strongest correlation with metabolic risks. The factor 2 (define as regional cardiac fat-CAD factor) explained the maximum variance (15.10 %), and EATth-LAVG is a most important contributor of developing coronary artery disease. Most importantly, it is postulated that global adipose tissue could contribute to metabolic syndrome and regional cardiac adipose tissue could contribute more to CAD. These observations were unique in this study never reported before. We used factor analysis to concurrently investigate the clustering of various measurements of adipose tissue that are thought to be important components of cardiometabolic risks. Two expected factors emerged explaining 65 % of the variance. The current study results clearly demonstrate, as far as we know for the first time, that different effects and correlation coefficient of global and regional fat distribution on cardiometabolic risk factors concurrently. The current study results suggest, as far as we know for the first time, that global fat depots could reflect a contribution to a systemic effect on metabolic syndrome while regional-specific cardiac fat depots may exert major local toxic effects on coronary artery disease. This finding is also supported by previous theory. And for the first time, this theory has been well addressed in different impacts of ectopic abdominal and cardiac fat deposits on cardiometabolic risks in a direct head-to-head comparison.

### Pathophysiology

Adipose tissue is a metabolically active endocrine organ that secretes and regulates multiple adipocytokines including leptin, Interleukin-6 (IL-6), tumor necrosis factor-α (TNF-α), resistin, adiponectin, monocyte chemoattractant protein-1 (MCP-1) and free fatty acids (FFA), and to have potential systemic or local effects on cardiometabolic risks, determined by differential distribution of ectopic fat deposits [5, 31–33]. The mechanism by which the different distribution of ectopic fat deposits associated with cardiometabolic risks is currently not well-established. Recent research has revealed that increased abdominal visceral fat accumulation provides excessive systemic free fatty acid and inflammatory cytokine in the portal vein, which accelerates insulin resistance and increased risk of metabolic syndrome [7, 34, 35].

In contrast to the excessive abdominal visceral fat depots with predominantly systemic metabolic effects, we found that regional-specific ectopic cardiac fat depots in EATth-LAVG, instead of total volume of EAT or PAT, is the most important parameter of EAT adiposity in prediction of atherosclerosis, independently even after adjustment for conventional risk factors and CAC score. Because EAT asymmetrically covers 80 % of the heart’s surface, especially in the coronary groove segments along the course of the epicardial coronary vessels, we could postulate that excessive regional cardiac fat deposits (especially in EATth-LAVG) may play a role in pathogenesis of CAD by diffusion of EAT paracrine metabolites through thin-walled coronary venous networks embedded in left AVG. and drainage into the coronary sinus and exert local toxic effects on coronary arteries atherosclerosis. This finding is also supported by our previous study and other studies [17, 21, 36].

### Strengths and limitation

This study has several strengths. First, this is the first comprehensive study to investigate different distribution of abdominal and cardiac fat (including total volume of AVAT, EAT, PAT and regional-specific cardiac fat simultaneously, and to compare the different impacts on cardiometabolic risks simultaneously. Second, we measured total volume of abdominal and cardiac adipose tissue, which might be more representative of global adiposity instead of measuring total fat area as described in previous studies [37, 38]. Third, we measured various fat parameter in the same subject at the same time in a direct head-to head comparison of the different abdominal and cardiac fat deposits with cardiometabolic risks, instead of indirect comparison in previous literature review.

Some limitation of this study must be taken into consideration. Due to the cross-sectional study design, the results, while showing correlations, do not imply causality between different ectopic fat depots and cardiometabolic risks, We did not measure parameters of insulin resistance or inflammation such as insulin sensitivity index or high-sensitivity C-reactive protein. Therefore, the mechanism of insulin resistance and global inflammation related to abdominal or cardiac visceral fat and cardiometabolic risks cannot be directly analyzed.

## Conclusion

In conclusion, total AVAT, EAT and PAT are associated with increasing number of metabolic syndrome components; while regional cardiac fat, especially EATth-LAVG, has stronger association with coronary atherosclerosis, measured by the severity of Agatston score. Our results suggest that global adiposity (especially AVAT) potentially has systemic inflammatory effect and contributes to the development of metabolic syndrome, while regional specific cardiac adiposity (especially measured by EATth-LAVG) may exert potentially local toxic effects on coronary arteries. Quantitative measurements of ectopic abdominal and regional-specific cardiac fat may potentially improve cardiometabolic risk assessment.

## Abbreviations

AIVG: Anterior interventricular groove
AVAT: abdominal visceral adipose tissue
BMI: Body mass index
CAC: Coronary artery calcification
CAD: Coronary artery disease
CT: Computed tomography
EAT: epicardial adipose tissue
FFA: free fatty acids
HDL-C: High-density lipoprotein-cholesterol
HOMA: Homeostatic model assessment
IL-6: interleukin-6
LAVG: left atrioventricular groove
MCP-1: Monocyte chemoattractant protein-1
PAT: paracardial adipose tissue
RAVG: right atrioventricular groove
TG: Triglyceride
TNF-α: Tumor necrosis factor-α
WC: waist circumference
